# Biopsy in emergency gastroscopy does not increase the risk of rebleeding in patients with Forrest I acute nonvariceal upper gastrointestinal bleeding combined with suspected malignant gastric ulcer: a multicenter retrospective cohort study

**DOI:** 10.1186/s12876-021-01836-z

**Published:** 2021-06-06

**Authors:** Quchuan Zhao, Tianyu Chi

**Affiliations:** Department of Gastroenterology, Xuanwu Hospital of Capital Medical University, 45 Chang-chun Street, Beijing, 100053 China

**Keywords:** Biopsy, Endoscopy, Rebleeding, Antithrombotic, Nonvariceal upper gastrointestinal bleeding

## Abstract

**Background:**

Few studies have reported whether a biopsy in emergency gastroscopy (EG) increased the risk of rebleeding in patients with Forrest I acute nonvariceal upper gastrointestinal bleeding (ANVUGIB) combined with suspected malignant gastric ulcer (SMGU). This study aims to conduct a multicenter retrospective cohort study using propensity score matching to verify whether a biopsy in EG increases the risk of rebleeding in patients diagnosed with Forrest I ANVUGIB combined with SMGU.

**Methods:**

Using the data for propensity-matched patients, logistic regression models were fitted using rebleeding as the dependent variable. Survival time was defined as the length of time the patient experienced from visiting the emergency department to rebleeding. We used the Kaplan–Meier (KM) method to analyze the 30-day survival of the patients with and without a biopsy after matching, and the log-rank test was performed to examine the differences in survival.

**Results:**

With the use of propensity score matching, 308 patients who underwent a biopsy in EG were matched with 308 patients who did not. In the five logistic regression models, there were no significant group differences in the risk of rebleeding in patients with Forrest I ANVUGIB combined with SMGU between the biopsy and no-biopsy groups. The probability of survival was not significantly different between the no-biopsy and biopsy groups.

**Conclusions:**

In this multicenter, retrospective propensity score matching cohort study, compared with patients without a biopsy, patients with a biopsy during EG had no increased risk of rebleeding, and there was no significant difference in the rate of rebleeding.

## Background

Acute nonvariceal upper gastrointestinal bleeding (ANVUGIB) is one of the most common acute critical diseases in clinical practice. The morbidity of ANVUGIB in Europe ranged from 25/100,000 to 35/100,000 in 2000, and the morbidity of ANVUGIB in the United States was 60.6/100,000 in 2009, among which the morbidity of ANVUGIB caused by peptic ulcers was 32.1/100,000 [1, 2]. Recently, a retrospective large-scale case analysis in China showed that compared with 1997–1998, peptic ulcer bleeding (52.7%) was still the most important cause of upper gastrointestinal bleeding (UGIB) in 2012–2013, the detection rate of high-risk ulcers (Forrest Ia, Ib, IIa and IIb) increased (28.2% vs.15.7%), and the overall mortality did not decrease significantly (1.7% vs. 1.1%) [3].

Emergency gastroscopy (EG) is an important method for the diagnosis and treatment of ANVUGIB [4]. For patients with ANVUGIB complicated with hemodynamic instability, EG should be performed as soon as possible to determine the cause after active fluid resuscitation [5]. The treatment of endoscopic hemostasis for Forrest Ia–IIb hemorrhagic lesions is also recommended [6]. However, the rebleeding risk of Forrest I hemorrhagic lesions is still high and can amount to 55% [5, 6]. Part of the reason for rebleeding was due to suspected malignant gastric ulcer (SMGU) [7].

The treatment of gastric ulcers combined with bleeding is completely different according to whether they are benign or malignant [8]. However, few studies have reported whether a biopsy in EG is necessary to determine the nature of Forrest I ANVUGIB combined with SMGU, and whether a biopsy increases the risk of rebleeding is still controversial [2, 7, 9].

This study conducted a multicenter retrospective cohort study using propensity score matching to verify whether a biopsy in EG increases the risk of rebleeding in patients diagnosed with Forrest I ANVUGIB combined with SMGU.

## Methods

### Data source and oversight

We searched the clinical data of all patients diagnosed with UGIB from June 2010 to June 2020 in the medical records system of three tertiary hospitals in Beijing, including diagnosis, treatment, vital signs, laboratory and imaging tests, EG results, pathology results, hospitalization costs, and demographic data. To protect the privacy of patients, the information related to the patient's name and identity was deleted in the search strategy. A unique reference number was allocated to each individual patient, facilitating data retrieval and further analysis. This study protocol was approved by the ethics committee of Xuanwu Hospital of Capital Medical University. Informed consent was waived as the data used in this study were anonymized, which was approved by the ethics committee at our hospital.

### Study design

We performed a retrospective cohort study. ANVUGIB combined with gastric ulcer was identified in the medical records system using physician-assigned International Classification of Diseases 10th revision (ICD-10) codes. We included possible diagnoses of ANVUGIB combined with gastric ulcer, such as K92.204 (upper gastrointestinal bleeding) plus K25 (gastric ulcer), K25.001 (acute gastric ulcer with bleeding), K25.301 (acute gastric ulcer) and K25.401 (gastric ulcer with bleeding).

Inclusion criteria: Patients were eligible for inclusion in the study if they (> 18 years old) were admitted to the emergency department (ED) between June 2010 and June 2020 with evidence of Forrest I ANVUGIB combined with SMGU, and if they did not take antithrombotic or took only one of the antithrombotic, such as aspirin or clopidogrel. The exclusion criteria were as follows: (1) esophagogastric variceal bleeding; (2) gastric carcinoma confirmed before EG; and (3) incomplete medical records.

### Forrest classification

Forrest classification was as follows: Ia spurting bleeding, Ib oozing bleeding, IIa nonbleeding visible vessel, IIb an adherent clot, IIc flat pigmented spot, and III clean base ulcer. The sites of bleeding were classified as the esophagus, stomach, and duodenum according to the gastroscopy results [10].

Some scoring systems have been developed to predict bleeding outcomes for patients with ANVUGIB bleeding. The Blatchford risk score is the most widely used scoring system in clinical practice (Table 1) [11, 12].

**Table 1:** Blatchford score

Indication	Score	Indication	Score
Blood urea, mmol/L		Systolic BP, mm Hg	
6.5–7.9	2	100–109	1
8.0–9.9	3	90–99	2
10.0–24.9	4	< 90	3
> 25	6	Other risk factors	
Hemoglobin, g/L, men		Pulse (≥ 100/bpm)	1
120–129	1	Melena	1
100–119	3	Syncope	1
< 100	6	Liver disease	2
Hemoglobin, g/dL, women		Heart failure	2
100–119	1	Maximum score^a^	23
< 100	6		

^a^A score ≥ 6 is classified as medium or high risk, and a score < 6 is classified as low risk

### The diagnostic criterion of rebleeding

Rebleeding was defined as one or more signs of ongoing bleeding, including hematemesis, melena, hematochezia, vital sign instability and a continuous drop in hemoglobin after the initial resuscitation or initial endoscopic therapy of the patient, which required repeated EG, angiographic embolization or operation to stop the bleeding [3, 12].

### Outcomes

The primary outcome of the study was the risk of hospital rebleeding the secondary outcome was hospitalization costs. These patients were followed throughout the hospital course until in-hospital rebleeding episodes.

### Sample size calculation

PASS 15 (NCSS, LCC., Kaysville, Utah) was used to calculate the sample size for the cohort study. operation procedure: Proportion → Two Independent Proportions → Test (Inequality) → Tests for Two Proportions (Ratios). According to guidelines, the rebleeding rate of Forrest I ANVUGIB is 55%, and we hypothesized that a biopsy has a low risk effect (RR = 1.2) at increasing rebleeding compared with no biopsy [9, 13]. To detect this difference with 80% power and a significance level of 0.05, 307 patients were considered necessary for each group.

### Statistical analysis

We used logistic and linear regression analyses after propensity score matching to control for confounding factors in this real-world study.

Given the differences in the baseline characteristics between eligible participants in the two groups (Table 2), propensity score matching was used to identify a cohort of patients with similar baseline characteristics. The propensity score is a conditional probability of having a particular exposure (a biopsy vs. no biopsy) given a set of baseline measured covariates. The propensity score was estimated with the use of a non-parsimonious multivariate logistic regression model, with biopsy as the dependent variable and all the baseline characteristics outlined in Table 2 as covariates. Matching was performed with the use of a 1:1 matching protocol without replacement (nearest-matching algorithm), with a caliper width equal to 0.2 of the standard deviation of the logit of the propensity score. Standardized differences were estimated for all the baseline covariates before and after matching to assess pre-match imbalance and postmatch balance. Standardized differences of less than 0.1 for a given covariate indicate a relatively small imbalance.

**Table 2:** Baseline characteristics before and after propensity score matching

Characteristic	Before matching			After matching		
	Biopsy (n = 401)	No-biopsy (n = 609)	Standardized difference	Biopsy (n = 308)	No-biopsy (n = 308)	Standardized difference
*Sex (%)*			0.0803			0.0032
Male	67.8%	75.9%		73.7%	74.0%	
Female	32.2%	24.1%		26.3%	26.0%	
*Age*			0.3042			0.0455
Distribution (%)						
≤ 59 years	44.1%	44.2%		50.3%	42.5%	
60–69 years	18.2%	35.0%		17.2%	30.5%	
70–79 years	14.7%	11.5%		14.9%	16.2%	
≥ 80 years	22.9%	9.4%		17.5%	10.7%	
*Emergency symptoms (%)*			0.1469			0.0617
Melena	27.2%	41.9%		35.1%	41.2%	
hematemesis	72.8%	58.1%		64.9%	58.8%	
*Forrest* classification (%)			0.2534			0.0552
Ib	28.7%	54.0%		37.0%	42.5%	
Ia	71.3%	46.0%		63.0%	57.5%	
Blatchford score	14.44 ± 2.80	13.50 ± 2.37	0.9414	13.92 ± 2.72	13.63 ± 2.27	0.0990
*Antithrombotics (%)*			0.1358			0.0899
Non-use	41.1%	44.3%		44.2%	43.5%	
Aspirin	41.9%	49.1%		37.3%	42.6%	
Clopidogrel	17.0%	6.6%		18.5%	13.9%	
*HP (%)*			− 0.0425			− 0.0422
Yes	61.6%	65.8%		63.3%	67.5%	
No	38.4%	34.2%		36.7%	32.5%	
*Pathology results*			0.1643			0.0584
Malignancy	40.4%	24.0%		36.0%	30.2%	
Benign	59.6%	76.0%		64.0%	69.8%	
*Ulcer diameter (%)*			0.3713			0.0422
< 1 cm	21.4%	44.0%		26.9%	38.3%	
1–1.9 cm	52.1%	43.2%		56.2%	42.2%	
2–2.9 cm	23.2%	10.5%		16.9%	14.9%	
≥ 3 cm	3.2%	2.3%		0.0%	4.5%	
*Transfusion (%)*			0.2341			0.0812
Yes	74.3%	50.9%		66.6%	58.4%	
No	25.7%	49.1%		33.4%	41.6%	
*Diabetes (%)*			− 0.0138			0.0357
Yes	28.2%	29.6%		30.5%	26.9%	
No	71.8%	70.4%		69.5%	73.1%	

Group differences were evaluated with Mann–Whitney U-test, Student’s t test, and χ^2^ or Fisher’s exact tests. Using the data for the propensity-matched patients, logistic regression models were fitted using rebleeding as the dependent variable. Using the data for the propensity-matched patients, multivariate linear regression models were fitted using hospitalization costs as the dependent variable.

A two-tailed P value < 0.05 was considered significant. All analyses were conducted using SPSS 23.0 (IBM Corp., Armonk, NY).

### Sensitivity analysis

To test the robustness of the main results, several additional analyses were conducted. First, multiple imputation using multivariate normal distribution was performed to evaluate the potential influence of missing data. Second, using the data for all the patients with Forrest I ANVUGIB combined with SMGU before matching, logistic regression models were fitted using rebleeding as the dependent variable. Third, using the data for all the patients with Forrest I ANVUGIB combined with SMGU before matching, multivariate linear regression models were fitted using hospitalization costs as the dependent variable. Fourth, subgroup analysis with the data before and after matching was also conducted by stratifying patients into the non-taking antithrombotic group, aspirin-alone group and clopidogrel-alone group.

### Survival analysis

Survival time was defined as the length of time the patient experienced from visiting the emergency department to rebleeding. We used the Kaplan–Meier (KM) method to analyze the 30-day survival without rebleeding of the patients with and without a biopsy after matching, and the log-rank test was performed to examine the differences in survival.

## Results

### Study population

**Fig. 1:**
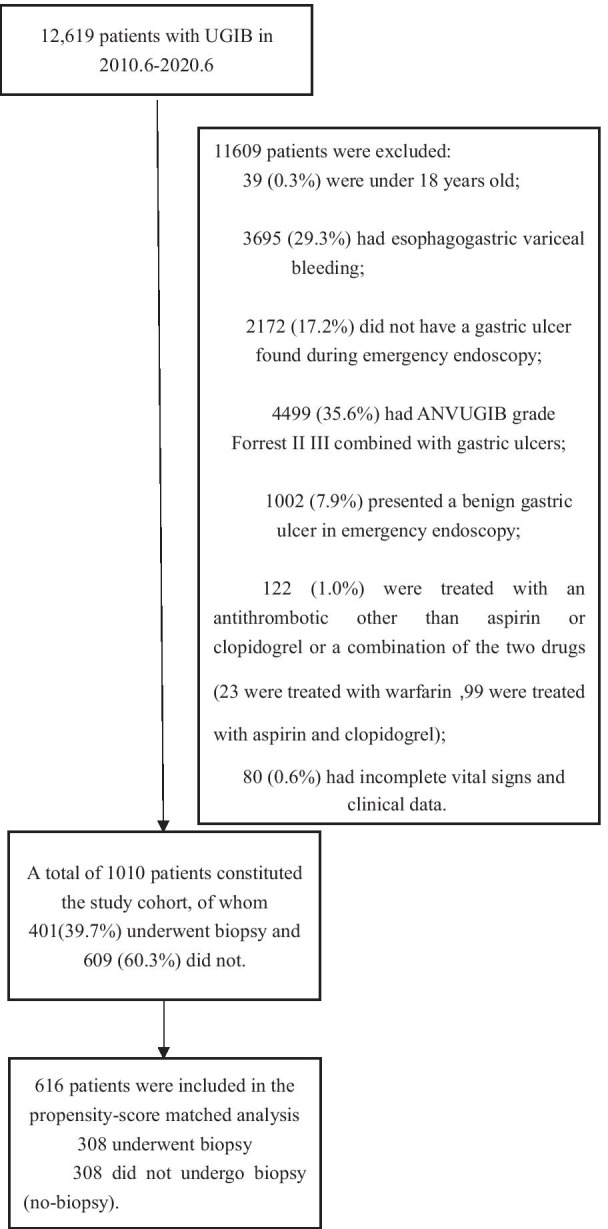
Flow chart of the identification of the study sample

Figure 1 illustrates the patient selection process. A total of 12,619 patients with UGIB who presented to the emergency department during the study period were identified. A total of 39 (0.3%) patients were less than 18 years old; 3695 (29.3%) had esophagogastric variceal bleeding; 2172 (17.2%) did not have gastric ulcers found during EG; 4499 (35.6%) were Forrest II III ANVUGIB combined with gastric ulcer; 1002 (7.9%) presented benign gastric ulcer in EG; 122 (1.0%) were treated with an antithrombotic other than aspirin or clopidogrel or a combination of the two drugs (23 were treated with warfarin and 99 were treated with both aspirin and clopidogrel); and the vital signs and clinical data of 80 (0.6%) were incomplete. Thus, these patients were excluded.

A total of 1010 patients constituted the study cohort, of whom 401 (39.7%) underwent a biopsy and 609 (60.3%) did not. Before propensity score matching, there were differences between the two groups in several of the baseline variables (Table 2). With the use of propensity score matching, 308 patients who underwent a biopsy in EG were matched with 308 patients who did not. After matching, the standardized differences were less than 0.1 for all variables, indicating only small differences between the two groups (Table 2).

### Primary outcome

In the cohort before and after matching, there were no significant differences in the risk of rebleeding in patients with Forrest I ANVUGIB combined with SMGU during EG between a biopsy and no biopsy (Table 3).

**Table 3:** Risk of primary outcomes in the cohort before and after propensity score matching

Outcome	Before matching				After matching			
	No. of Patients with Event	Event Rate (%)	Hazard Ratio (95% CI)	P Value	No. of Patients with Event	Event Rate (%)	Hazard Ratio (95% CI)	P Value
Rebleeding				0.077				0.548
biopsy	258/401	64.3%	1.09 (0.99–1.21)		204/308	66.2%	0.97 (0.87–1.08)	
No-biopsy	358/609	58.8%	Reference		211/308	68.5%	Reference	

Table 4 summarizes the outcomes according to biopsy and no biopsy before and after matching. In the five logistic regression models, there were no significant group differences in the risk of rebleeding in patients with Forrest I ANVUGIB combined with SMGU.

**Table 4:** Logistic regression analysis (biopsy group vs. no-biopsy group) in the cohort before and after propensity score matching

	Before Matching Rebleeding			After Matching Rebleeding		
	Odds ratio	95% CI	P value	Odds ratio	95% CI	P value
Unadjusted	1.265	0.975–1.641	0.077	0.902	0.644–1.263	0.548
Adjusted for all covariates^a^	1.028	0.754–1.400	0.862	0.834	0.573–1.214	0.343
Multiple imputation^b^	1.040	0.772–1.400	0.798			

^a^All variables in Table 2 were included as covariates for the model with hospital rebleeding

^b^Logistic regression was performed including all of the patients before matching after multiple imputation for missing data using multivariate normal distribution

### Hospitalization costs

In the cohort before matching, there was no significant difference in the hospitalization costs of patients with Forrest I ANVUGIB with SMGU during EG between biopsy and no biopsy. In the cohort after matching, the hospitalization costs of patients undergoing a biopsy with Forrest I ANVUGIB combined with SMGU during EG were significantly lower than those of patients who did not undergo a biopsy (Table 5).

**Table 5:** Hospitalization costs in the cohort before and after propensity score matching

Outcome	Before matching	P Value	After matching	P Value
Hospitalization costs(*10^4^RMB)		0.157		0.045
Biopsy	6.24 ± 4.83		5.76 ± 4.77	
No-biopsy	5.79 ± 5.03		6.57 ± 5.32	

Table 6 summarizes the outcomes according to biopsy vs. no biopsy before and after matching. In the cohort before and after matching, multiple linear regression analysis showed that a biopsy was a beneficial factor in significantly reducing hospitalization costs.

**Table 6:** Multivariate linear regression analysis (biopsy group vs. no-biopsy group) in the cohort before and after propensity score matching

	Before matching Hospitalization costs			After matching Hospitalization costs		
	B	95% CI	P value	B	95% CI	P value
Unadjusted	0.447	− 0.178 to 1.072	0.161	− 0.817	− 1.616 to − 0.017	0.045
Adjusted for all covariates^a^	− 1.476	− 1.629 to − 1.324	< 0.001	− 1.424	− 1.584 to − 1.263	< 0.001
Multiple imputation^b^	− 1.481	− 1.602 to − 1.361	< 0.001			

^a^All variables in Table 2 included as covariates. For model with Hospitalization costs

^b^Multivariate linear regression was performed for all the patients before matching after multiple imputation for missing data using multivariate normal distribution

### Sensitivity analysis

The multiple imputation presented a familiar consequence: there were no significant differences in the risk of rebleeding in patients with Forrest I ANVUGIB combined with SMGU during EG between biopsy and no biopsy (Table 4); a biopsy was a beneficial factor in significantly reducing hospitalization costs (Table 5).

We also performed statistical analysis on all patients before matching and obtained similar results. There were no significant differences in the risk of rebleeding in patients with Forrest I ANVUGIB combined with SMGU during EG between biopsy and no biopsy groups (Table 3). A biopsy is a beneficial factor to significantly reduce the costs of hospitalization (Table 6).

Subgroup analysis with the data before and after matching was also conducted by stratifying patients into the non-taking antithrombotic group, aspirin-alone group and clopidogrel-alone group. In all subgroup analyses, there were no significant differences in the rate of rebleeding between patients who underwent a biopsy in EG and those who did not (Table 7).

**Table 7:** Subgroup logistic regression analysis (clopidogrel group vs. aspirin group vs. non-use group) in the cohort before and after propensity score matching

	Before matching Rebleeding			After Matching Rebleeding		
	Odds ratio	95% CI	P Value	Odds ratio	95% CI	P Value
*Unadjusted*						
Non-use	Reference			Reference		
Aspirin	1.144	0.875–1.496	0.324	1.022	0.714–1.462	0.905
Clopidogrel	0.988	0.644–1.518	0.958	1.048	0.600–1.828	0.870
*Adjusted for all covariates*^*a*^						
Non-use	Reference			Reference		
Aspirin	0.673	0.446–1.016	0.059	0.757	0.480–1.194	0.231
Clopidogrel	0.740	0.446–1.227	0.243	0.735	0.373–1.449	0.374

^a^All variables in Table 2 were included as covariates for the model with hospital Rebleeding

### Survival analysis

**Fig. 2:**
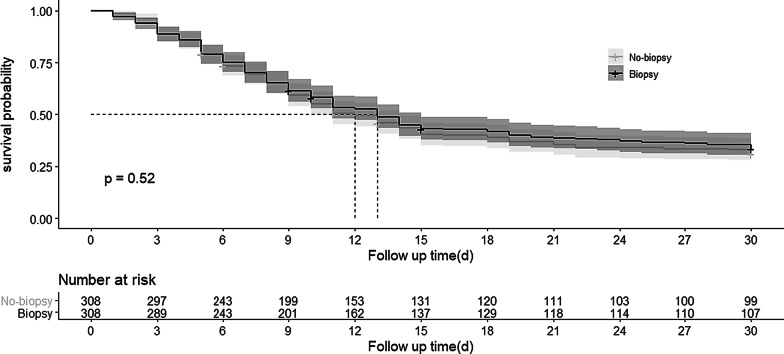
Kaplan–Meier (KM) curve for 30-day survival without rebleeding for patients after matching

Figure 2 demonstrates a KM curve for 30-day survival for the patients with and without a biopsy after matching. The probability of survival was not significantly different between the no-biopsy and biopsy groups (P = 0.520 by log-rank test).

### Discussion

To our knowledge, this is the first multicenter retrospective cohort study that focused on biopsies during EG in patients with Forrest I ANUGIB combined with SMGU using a propensity-matched approach. The present results demonstrated that there were no significant differences in the risk of rebleeding in patients with Forrest I ANVUGIB combined with SMGU during EG between biopsy and no-biopsy groups. The hospitalization costs of patients undergoing a biopsy with Forrest I ANVUGIB combined with SMGU during EG were significantly lower than those of patients who did not undergo a biopsy.

ANVUGIB accounts for 80%–90% of UGIB, with peptic ulcers, acute gastric mucosal injury and upper gastrointestinal tumors as the most common causes [2]. In recent years, with the widespread use of antithrombotic, such as aspirin and clopidogrel, the amount of ANVUGIB caused by these drugs has increased year by year [14, 15]. The guidelines recommend that for ANVUGIB, a biopsy should be performed under direct vision to determine the nature of lesions found during EG, where malignant lesions are suspected as long as circumstances permit [3, 16]. However, patients with ANVUGIB requiring EG are often more complex, and the guidelines do not specify the circumstances under which biopsy of a suspected malignant lesion is performed, a situation in which the clinician mostly operates on the basis of their own clinical experience [3, 12, 16]. Clinicians often encounter the following difficulties in making decisions [17–20]: (1) Can biopsy be performed in patients with active bleeding under EG? (2) What are the levels of Forrest classification for active bleeding during EG that can be biopsied? (3) Can biopsy be performed in patients who have taken antithrombotic within 24 h before EG? (4) Under these circumstances, compared with patients without biopsy during EG, do patients undergoing biopsy have an increased risk of rebleeding? (5) Is there any difference between the hospitalization costs of patients with timely diagnosis of lesions through biopsy during EG and those without biopsy? These questions have not been clarified in the guidelines or in previous studies, but our study exactly answers the above questions.

To answer these most difficult questions that clinicians encounter in EG, we selected patients with Forrest I (the level with the highest risk of rebleeding) ANVUGIB to constitute the cohort. Active bleeding in the lesion can be seen during gastroscopy in patients with Forrest I ANVUGIB, and the patient is in a critical situation and must undergo endoscopic hemostatic therapy [16, 21]. However, whether biopsy is required to identify the nature of suspected malignant lesions after endoscopic hemostasis during emergency gastroscopy has not been clarified in the guidelines and consensus, which is also a controversial issue in clinical practice [3, 22]. To our knowledge, conventional drug treatment for malignant lesions is often ineffective, and patients are prone to recurrent and life-threatening bleeding, so surgical treatment is often the best treatment option for such patients [8]. Our study clarifies these controversies precisely by showing that univariate and multivariate analyses before and after matching had no increased risk of rebleeding and no statistically significant difference in the rate of rebleeding in patients with Forrest I ANVUGIB combined with SMGU who underwent biopsy compared with those who did not in EG. In the emergency endoscopic examination and treatment of these critical patients, biopsy not only does not increase the risk of rebleeding but also clarifies the nature of the lesions, providing strong support for the surgical treatment of ANVUGIB caused by malignant lesions.

Our study showed that in univariate analysis after matching and multivariate analysis before and after matching, the hospitalization costs of patients undergoing biopsy with Forrest I ANVUGIB combined with SMGU during EG were significantly lower than those of patients who did not undergo a biopsy. According to the guidelines and consensus recommendations, benign and malignant lesions determine the difference in the treatment paths of patients with ANVUGIB [12]. Timely determination of the nature of bleeding lesions during EG by a biopsy provides strong support for clinicians to choose the correct clinical path and to avoid the mistake of treatment decision-making [3]. For malignant lesions, timely and effective surgical operations can avoid the expense of excessive drug treatment and thus save hospitalization costs [23, 24]. However, in univariate analysis before matching, there was no significant difference in the hospitalization costs of patients undergoing a biopsy during EG with Forrest I ANVUGIB combined with SMGU and those without. This outcome was considered to be related to the high Blatchford score of patients in the biopsy group before matching as many studies have shown that Blatchford score is positively correlated with the hospitalization costs [25, 26].

According to the guidelines and consensus, in routine gastroscopy, a biopsy is a low-risk operation, aspirin- or clopidogrel-alone can be continued before a biopsy, and there is no significant difference in the rate of rebleeding with the withdrawal of antithrombotic [9, 13, 17]. However, the guidelines and consensus did not clarify whether ANVUGIB patients who had taken aspirin or clopidogrel alone within 24 h before the examination could be biopsied in EG, whether the risk of rebleeding was increased by a biopsy compared with those who did not, and whether there was a significant difference in the risk of rebleeding [27, 28]. Our study also answers these questions. We conducted subgroup analysis and illustrated that in univariate and multivariate analyses before and after matching, patients undergoing a biopsy during EG with Forrest I ANVUGIB combined with SMGU in the non-taking antithrombotic, aspirin-alone, and clopidogrel-alone groups had no increased risk of rebleeding and no significant differences in the risk of rebleeding when compared with patients who did not.

Some limitations of our analysis should be considered. First, this was a nonrandomized, observational study and hence suffers from potential selection and ascertainment bias, despite robust propensity score matching. Second, we did not compare the risk of death between the biopsy and the no-biopsy groups because ANVUGIB has a mortality rate of 1.1% to 1.7%, and our current sample size does not support a comparison of the risk of death. In our cohort, before matching, there were 6 deaths (6/401 1.5%) in the biopsy group and 8 deaths (8/609 1.3%) in the no-biopsy group; after matching, there were 3 deaths (3/308 1.0%) in the biopsy group and 5 deaths (5/308 1.6%) in the no-biopsy group. In the future, we will continue to include data from more centers to have a sufficient sample size to analyze the risk of death.

## Conclusions

In this multicenter, retrospective propensity score matching cohort study of patients with Forrest I ANVUGIB combined with SMGU, compared with patients without a biopsy, patients with a biopsy during EG had no increased risk of rebleeding, and there was no significant difference in the rate of rebleeding. Patients with a biopsy had significantly lower hospitalization costs than those without a biopsy.

## Data Availability

The dataset generated and analyzed during the study is stored in a secure localized database but is available from the corresponding author in an anonymous format on reasonable request.

## Abbreviations

ANVUGIB: Acute nonvariceal upper gastrointestinal bleeding
EG: Emergency gastroscopy
SMGU: Suspected malignant gastric ulcer
KM: Kaplan–Meier
UGIB: Upper gastrointestinal bleeding
